# Isolation and Evaluation of Potential Use of Prebiotics—Utilizing Butyrate-Producing Bacteria in *Nibea coibor*

**DOI:** 10.1155/anu/4679037

**Published:** 2025-01-11

**Authors:** Zhongzhen Li, Ngoc Tuan Tran, Ming Zhang, Zhaoxi Li, Wanying Yang, Shuqi Wang, Zhong Hu, Shengkang Li

**Affiliations:** ^1^Guangdong Provincial Key Laboratory of Marine Biology, Shantou University, Shantou 515063, China; ^2^Institute of Marine Sciences, Shantou University, Shantou 515063, China; ^3^STU-UMT Joint Shellfish Research Laboratory, Shantou University, Shantou 515063, China; ^4^Zhuhai Institute of Translational Medicine, Zhuhai People's Hospital (Zhuhai Clinical Medical College of Jinan University), Jinan University, Zhuhai 519000, China; ^5^Guangdong Provincial Key Laboratory for Green Agricultural Production and Intelligent Equipment, College of Biological and Food Engineering, Guangdong University of Petrochemical Technology, Maoming 525000, China

**Keywords:** butyrate-producing bacteria, growth, gut microbiota, immune response, *Nibea coibor*, short-chain fatty acids

## Abstract

Butyrate-producing bacteria (BPB) benefit the health of aquatic animals. This current study aimed to isolate BPB from the intestines of *Nibea coibor* and assess their probiotic potential. The results showed that nine isolates were obtained in vitro from the gut of *N. coibor*, including six *Clostridium butyricum*, two *Proteocatella sphenisci*, and one *Fusobacterium varium*. The representative bacteria, *C. butyricum* CG-3 and *P. sphenisci* DG-1, which produce high butyrate levels, were further studied for short-chain fatty acid (SCFA) production and antibiotic susceptibility. The effects of BPB singly (CB: basal diet + CG-3 and PS: basal diet + DG-1, at 10^7^ CFU/g) or in combination with galactooligosaccharides (GOS) (0.5%) and inulin (0.5%) (CBIG) or D-sorbitol (0.5%) (PSGS) on the growth and health status of *N. coibor* were investigated. Results showed an increase in growth parameters in the CB, CBIG, and PSGS groups, except for the PS group. Alterations in intestinal microbiota (including diversity, abundance, and function) were observed in four experimental groups (CB, CBIG, PS, and PSGS groups). SCFA contents increased in treated groups; butyrate production was positively related to bacterial abundance. Compared to control, levels of complement C3, complement C4, immunoglobulin M (IgM), transforming growth factor-*β* (TGF-*β*), interleukin (IL)-10, IL-1*β*, and lysozyme (LZM) increased, while malondialdehyde (MDA) decreased in treated groups. Contents of IL-6 (PS and PSGS groups), tumor necrosis factor-alpha (TNF-*α*) (CB, PS, and PSGS groups), total antioxidant capacity (T-AOC) (CB and PS groups), total superoxide dismutase (T-SOD) (PS group), catalase (CAT) (CB and PSGS groups), and activities of amylase (PS and PSGS groups), trypsin (CB group), and lipase (CBIG group) were increased. Our results suggested the potential use of *C. butyricum* CG-1 or *P. sphenisci* DG-1 singly or in combination with prebiotics improved growth and health conditions in *N. coibor*.

## 1. Introduction

The gastrointestinal tract provides an optimal environment for microorganisms to colonize and grow. The microbiota can inhabit the epithelial surface or microvilli, or pass through the intestines as heterocysts or transient to be important in food digestion, nutrient and enzyme contribution, and immunity activation [1]. The gut microorganisms considerably affect the health condition of their hosts by generating metabolic products [2–4]. Commensal bacteria can ferment carbohydrates to form short-chain fatty acids (SCFAs) [2]. Among these, butyrate provides energy to epithelial cells, protects a healthy epithelial barrier, and participates in anti-inflammatory activities [5–7].

Currently, the contribution of butyrate to changes in physiology and immune system in animals has been widely studied [8–11]. In aquatic animals, butyrate has been reviewed to be important in regulating host immunity [12]. Dietary administration of butyric acid and its salt forms has shown significant effects on enhancing immune responses in both fish and shellfish [13]. In *Dicentrarchus labrax*, dietary butyrate supplementation showed positive effects on mucosa and immune homeostasis [14]. Also, in *Sparus aurata*, butyrate supplementation showed an increased survival of fish after bacterial infection with *Photobacterium damselae* subsp. *piscicida* prevented the low growth of fish infected with parasites, increased the diversity of intestinal bacteria, especially butyrate-producing bacteria (BPB), and protected gut mucosal proteome caused by vegetable diets [15]. Nevertheless, the application of salts and other forms of organic acids in fish needs to be considered because of differences in the basic anatomical and physiological characteristics between fish species and other terrestrial animals [16]. More specifically, in fish, issues related to the intestinal length, feed passage duration through the intestine, and the resistance of acid/organic salts in feed to hydrochloric acid in the stomach are given consideration [16]. Notably, the application of BPB as an alternative approach to promote butyric acid production in fishes has received notice from researchers (reviewed by Tran et al. [17]). For example, in aquaculture, BPB (i.e., *Clostridium butyricum*) shows benefits for survival, growth, digestion, intestinal structures, gut microbiota, immune system, and metabolic function [18–21]. Therefore, BPB are demonstrated to provide effectiveness in the health status of fishes.

Probiotics maintain host health if administered in sufficient quantities of bacterial cells [22]. In aquaculture, probiotics have positive effectiveness in contributing nutrients, improving growth and immune responses, and preventing disease occurrence [23]. For example, the lactic acid bacteria, *Enterococcus faecalis* YFI-G720 (at 10^6^ CFU/g), enhanced growth, serum C3, and immunoglobulin M (IgM), relative mRNA expression of C3, IgM, and interleukin (IL)-1*β*, and resistance ability to *Aeromonas veronii* infection, and altered intestinal microbiota in *Carassius auratus* [24]. In a previous study, the application of a probiotics supplementation diet improved the physiological, digestive, antioxidant, and immune profile in various fish against different pathogens [25–29]. The probiotic application has been considered one of the effective strategies used for aquaculture [30].

Interestingly, butyrate formation is directly associated with the growth of BPB through the anaerobic fermentation of carbohydrates [31]. The combination of probiotics and prebiotics in fish feed has been shown to enhance growth, physiological–chemical variables, digestive-antioxidant status, and immunity level of different fishes to pathogens [32, 33]. In Chu's croaker (*Nibea coibor*), a previous study has demonstrated that the dietary mixture of inulin, galactooligosaccharides (GOS), and D-sorbitol significantly improved the growth, antioxidant, and immunity capacity changed the composition of the intestinal microbiome and promoted SCFA production [34]. Although the selective growth of BPB by dietary supplementation of carbohydrates has been reported, the identification of specific bacteria contributing to butyrate synthesis from aquatic animals remains unclear. Previously, in *Scylla paramamosain*, the contents of SCFAs (especially butyrate) and the proliferation of BPB were recorded by in vitro fermentation [12]. To date, in humans, research using traditional culture methods has obtained major groups of BPB [3, 31]. In the intestines of *Macrobrachium rosenbergii*, the previous study isolated a BPB strain, *C. butyricum* [35]. Surprisingly, in aquaculture, most BPB (i.e., *C. butyricum*) are isolated from other sources, i.e., chicken intestine, soil, and fish diet [36], but not from aquatic animals. Therefore, the selection of novel BPB isolated from aquatic animals is necessary to provide a probiotic source for used as a feed additive in aquaculture.

Chu's croaker (*N. coibor*) belongs to the suborder Percoidei, the family Sciaenidae, and the genus *Nibea* [37]. It is an economically important marine species, widely distributed across Japan, Korea, India, and Southeast China [38]. In addition to its delicious meat, high tolerance, and rapid growth rate, this species also possesses a large swim bladder, which is utilized in the production of high-quality fish glue (dried swim bladder, 10,000–15,000 RMB/kg) [38]. These characteristics make it a promising candidate for aquaculture in China. Our previous studies have identified a relationship between diets and potential SCFA-producing bacteria in the intestines of *Nibea* [39]. Furthermore, prebiotics may enhance growth performance, antioxidant capacity, and immune responses in *N. coibor* by modulating the intestinal microbiota composition and promoting subsequent SCFA production [34]. In this study, we successfully isolated two species of BPB capable of producing butyrate from the intestines of *N. coibor*. Carbohydrates can serve as substrates for BPB, enhancing their activity and supporting better digestion and nutrient utilization. Therefore, we also investigated the effects of BPB alone or combined with carbohydrates on growth, physiological characteristics, and intestinal microbiota of *N. coibor*. This study proved the applicability of BPB as a feed additive in *N. coibor*. The methodologies and findings could potentially be applied to various other fish species.

## 2. Materials and Methods

### 2.1. Anaerobic Fermentation Culture

Eight kinds of carbohydrates, including D-mannitol (S11072), D-sorbitol (S11057), GOS (S11138), inulin (S11143), L-sorbose (S11056), mannanoligosaccharides (MOS, S30907), xylitol (S11039), and xylooligosaccharides (XOS, S11137), were purchased from Yuanye Biotechnology Co. Ltd. (Shanghai, China), and used in the present study. Nine healthy *N. coibor* collected from a hatchery (Raoping Town, Guangdong, China) were used to collect gut contents. All fish were anesthetized with eugenol (1:10,000, Shanghai Reagent), rinsed with distilled water to remove surface contaminants, dried, and then sprayed with 75% ethanol before being transferred to a clean bench. The fish were aseptically dissected to remove the entire gastrointestinal tract, and its contents were squeezed into a sterilized centrifuge tube. The gut contents were deoxygenated with AnaeroPack (MGC, Tokyo, Japan) and kept at 4°C. The in vitro anaerobic screening was conducted within an anaerobic chamber supplied with 85% N_2_, 10% CO_2_, and 5% H_2_ at 28°C. The gut contents of three fish were pooled and anaerobically homogenized in sodium phosphate buffer (1% w/v), and the homogenates were filtrated to remove food residues. A volume of 2 mL of homogenates was transferred to sterile glass tubes supplemented with 0.01 g of D-mannitol, L-sorbose, xylitol, D-sorbitol, inulin, GOS, MOS, or XOS, or without carbohydrate (controls). The tubes were carefully sealed and shaken at 28°C for 36 h. After incubation, SCFAs in cultures were analyzed by GC6890N gas chromatography (Agilent Technologies, USA) [34].

### 2.2. Isolation and Identification of BPB

Three kinds of carbohydrates (D-sorbitol, GOS, and inulin) promoting the high production of butyrate after in vitro fermentation were used as substrates for the selective growth of BPB (Figure S1). Fresh gut contents of nine healthy *N. coibor* were collected. The 10% (w/v) intestinal culture supplemented with 0.5% D-sorbitol, GOS, or inulin was incubated at 28°C for 36 h under O_2_-free N_2_. Subsequently, 100-μL aliquots of each culture (at 10^−5^ and 10^−6^ serial dilutions) were spread onto the surface of an agar dish containing peptone-yeast extract (PY) [3], PYL (PY added with 0.5% D-sorbitol, GOS, or inulin), PYA (PY added with 33 mM sodium acetate), or PYAL (PYA added with 0.5% D-sorbitol, GOS, or inulin). The dishes were then anaerobically maintained at 28°C for 72 h. Morphologically distinct colonies on carbohydrate-containing media were selected to culture into PYAL broth medium at 28°C overnight. Butyrate production in the cultures was analyzed using GC6890N gas chromatography, and the colonies producing butyrate (>2 mM) were implied as BPB [31].

Isolated bacteria were identified through DNA sequencing. Briefly, bacterial DNA was isolated using a test kit (Cat# 4992448, Tiangen, China). The DNA was transferred to a commercial company to amplify and sequence the 16S rRNA gene. The 16S rRNA gene (~1500 bp) was amplified using the bacterial universal primers 27F and 1492R, according to the method described by Lane [40]. Identification of bacteria was performed based on Blastn search on NCBI. Phylogenetic trees were constructed using 16S rDNA sequences with the Neighbor-Joining method in MEGA 5.2.1 (AZ, USA).

Among the identified bacterial strains, the two representative BPB isolates, *C. butyricum* CG-3 and *Proteocatella sphenisci* DG-1, which produced significant amounts of butyrate as measured in this study, were selected for further analyses. Bacterial morphology was observed using a scanning electron microscope (JSM-6360LA, Jeol, Japan) after gold coating.

The growth and SCFA production capacity of the two strains were assessed. *C. butyricum* CG-3 was cultured in PYIG medium (PY broth containing PY, inulin, and GOS), while *P. sphenisci* DG-1 was cultured in PYGS medium (PY broth containing PY, GOS, and D-sorbitol). Every 4 h during bacterial growth, 2.5 mL of bacterial culture was transferred into two centrifuge tubes. Of this, 2 mL was used for SCFA detection, and the remaining volume was centrifuged at 5000 rpm for 5 min to harvest the cells. The bacterial cells were resuspended in sterile saline, and 200 µL of the suspension was added to a well of a 96-well plate to measure OD600. Three replicates were performed for each strain to generate growth curves.

### 2.3. Antibiotic Susceptibility of BPB

The representative BPB isolates, *C. butyricum* CG-3 and *P. sphenisci* DG-1, were used for further investigation. The disc diffusion method was applied to this experiment. A total of 20 antimicrobial agents, including amikacin (30 μg), ampicillin (10 μg), carbenicillin (100 μg), cefalexin (30 μg), cefazolin (30 μg), cefoperazone (75 μg), cefradine (30 μg), ceftriaxone (30 μg), ceftazidime (30 μg), cefuroxime (30 μg), doxycycline (30 μg), erythromycin (15 μg), gentamicin (10 μg), kanamycin (30 μg), minocycline (30 μg), neomycin (30 μg), oxacillin (1 μg), penicillin (10 U), piperacillin (100 μg), and tetracycline (30 μg), were used. PYA plates were spread with bacterial suspension (100 μL), added antibiotic discs to the surface, and maintained at 28°C. The culture was carried out for 24 h; the inhibition zone of each disc was measured and utilized for evaluating resistant, intermediate, or sensitive indices according to the manufacturer's recommendation.

### 2.4. Feeding Trial and Sampling

The basal diet, formulated with moisture (12%), crude protein (45%), crude lipid (9%), and ash (14%), was purchased from Yuequn Ocean Co., Ltd. (Guangdong, China). Five diets, comprising basal diet, CB (basal diet+*C. butyricum* CG3 at 10^7^ CFU/g), CBIG (CB + 0.5% inulin + 0.5% GOS), PS (basal diet + *P. sphenisci* DG1 at 10^7^ CFU/g), and PSGS (PS + 0.5% GOS + 0.5% D-sorbitol) were prepared and used for supplementation feeding the fish. The selected strains, *C. butyricum* CG3 and *P. sphenisci* DG1, were cultured in PYIG/PYGS broth at 28°C for 24 h, harvested by centrifugation, and washed twice with sterile saline. The final dose of bacteria or carbohydrates (including inulin, GOS, and D-sorbitol) was diluted with normal saline and sprayed uniformly onto the basal diet. The feed was kept at 40°C for 12 h and transferred to 4°C for storage. The basal diet supplemented with normal saline was designed for the control group. All diets were reprepared weekly to ensure bacterial survival.

Three hundred juvenile *N. coibor* were raised at Nan'ao Marine Biological Station (Shantou University) for 2 weeks. Following acclimation, the fish grew to 13.59 ± 0.07 g and were randomly distributed into 15 floating cages measuring 1.0 m × 2.0 m × 1.5 m placed in the sea. According to a randomized block design, each cage accommodated 20 fish, with three cages randomly assigned to each of the following diets: control, CB, CBIG, PS, and PSGS. The weight of the fish was measured once a week during the culture period. All fish were fed (appropriately 3% body weight) two times daily (06:00 and 18:00) with corresponding diets. Environmental parameters (temperature 20–30°C, salinity ~36‰, DO >5.5 mg/L, and pH 8.7–8.9) were determined during the feeding period. After 8 weeks, fish survival rate (SR) was recorded; fish weight was noted to assess weight gain (WG), specific growth rate (SGR), and feed conversion ratio (FCR) [34].

Eight fish were collected from each cage, with each treatment consisting of three cages, totaling 24 fish. Blood samples were collected from the caudal vein using a 1 mL syringe, then maintained at 4°C for 4 h before being centrifuged at 3500 rpm for 20 min to obtain serum. After blood collection, the fish were rinsed with distilled water to remove surface contaminants, dried, and then sprayed with 75% ethanol before being transferred to a clean bench. The fish were aseptically dissected to remove the entire gastrointestinal tract, and its contents were squeezed into a sterilized centrifuge tube. The foregut was then cut and transferred to another sterilized centrifuge tube. In each treatment, samples from six out of 24 fish were pooled together to obtain a sufficient quantity for testing, and each treatment consisted of four replications. The samples were stored at −80°C for later analysis.

### 2.5. Gut Microbiota Analysis

Samples of intestinal contents were obtained and stored at −80°C under aseptic conditions. DNA from gut contents was extracted following the previous methods [34]. DNA was sent to a commercial company to amplify the 16S rRNA V4 region and to sequence the genes on the IonS5TMXL platform (Thermo Fisher Scientific, USA).

### 2.6. Microbial Fermentation Analysis

The contents of SCFAs were analyzed following the previous method [41, 42]. We mixed the intestinal contents in 2-ethylbutyric acid (internal standard) (1:9, w/v), with the pH value adjusted to 2–3 using H_2_SO_4_. The suspension was thoroughly mixed and centrifuged (13,000 rpm, 10 min) to achieve a clear liquid, which was filtered (using 0.2 μm filters) and utilized to measure the concentrations of SCFAs using a GC6890N gas chromatography (Agilent Technologies, USA).

### 2.7. Analysis of Digestive Enzymes, Antioxidant Capacity, and Innate Immunity

Lysozyme (LZM) activity was analyzed by a test kit manufactured by Nanjing Jiancheng Bioengineering Institute (Jiangsu, China). The contents of IgM, complement C3, complement C4, IL-1*β*, IL-10, IL-6, transforming growth factor-*β* (TGF-*β*), and tumor necrosis factor-alpha (TNF-*α*) were assayed using ELISA kits for fish (Shanghai Yanhui Bio-Technique Co. Ltd.).

The samples of foregut were suspended in saline (1:9, w/v) and centrifuged (3000 rpm, 15 min, 4°C) to obtain the clear liquid. The liquid was utilized to assess the levels of total superoxide dismutase (T-SOD), catalase (CAT), total antioxidant capacity (T-AOC), malondialdehyde (MDA), amylase, lipase, and trypsin using test kits (Nanjing Jiancheng Bioengineering Institute).

### 2.8. Bioinformatics

The read data were identified following their sequences with unique barcode sequences. Operational taxonomic units (OTUs) were obtained from sequences using Uparse (V7.0.1001). Classification analysis was carried out by the SILVA database (with >80% confidence). The *α*-diversity (based on Shannon and Simpson indices) was analyzed by QIIME (V1.7.0). The heatmap was prepared by performing OmicShare tools (www.omicshare.com/tools). The principal coordinates analysis (PCoA) was analyzed using VEGAN.

### 2.9. Statistical Analyses

One-way ANOVA was utilized to assess differences in data obtained between groups, performed by GraphPad Prism (Version 6, USA). The normality of the data and homogeneity of variance were tested before performing the ANOVA. Regression analysis was analyzed using GraphPad Prism. Statistically significant differences were confirmed at *p* < 0.05. Discriminatory bacterial groups in different groups were analyzed using LEfSe (with linear discriminant analysis [LDA] >3.5).

## 3. Results

### 3.1. SCFA Formation in the In Vitro Anaerobic Fermentation

**Figure 1:**
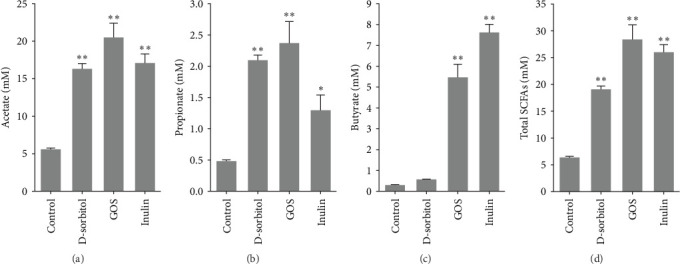
Concentrations of SCFAs after 36 h of fermentation of three kinds of carbohydrates in the *in vitro* cultures (*n* = 3): (A) acetate production, (B) propionate production, (C) butyrate production, and (D) total SCFA production. Asterisks indicate significant differences from the control (*⁣*^*∗*^*p* < 0.05 and *⁣*^*∗∗*^*p* < 0.01) by one-way ANOVA with Dunnett's multiple comparisons test. Values are presented as mean ± SEM. GOS, galactooligosaccharides; SCFA, short-chain fatty acid.

The amount of butyrate was higher in groups supplemented with inulin, D-sorbitol, or GOS than in others in the in vitro fermentation (Figure S1). The positive effect of inulin, D-sorbitol, or GOS on SCFA production was further confirmed. A significant increase in SCFA production was observed in all cultures, except nonsignificant butyrate production in the D-sorbitol supplementation group was observed (Figure 1).

### 3.2. Identification and Characterization of BPB

Fifty-seven colonies were selected from plates containing PY/PYA supplemented with D-sorbitol (8/10 colonies, respectively), GOS (8/8 colonies, respectively), or inulin (11/12 colonies, respectively). Among these colonies, one isolated from D-sorbitol, six from GOS, and two from inulin showed butyrate-producing activity in both PYL (PY broth supplemented with 0.5% D-sorbitol, GOS, or inulin) and PYAG (PY broth supplemented with 33 mM sodium acetate and 1% glucose) media (Table 1). Six isolates (named strains CI−1, CI−2, and CG−1, −2, −3, and −4) were closely related to *C. butyricum* (with 99% identity), two isolates (DG-1 and CS-1) were *Proteocatella sphenisci* (98% identity), and one isolate (CG-5) was *Fusobacterium varium* (99% identity). The genetic relationship among these bacterial isolates was illustrated by a phylogenetic tree (Figure S2). Among the identified bacteria, two isolates, *C. butyricum* CG-3 and *P. sphenisci* DG-1, produced marked amounts of butyrate in PYAG broth, with concentrations of 26.50 ± 0.85 mM and 27.76 ± 0.28 mM, respectively, and were selected for further investigations.

**Figure 2:**
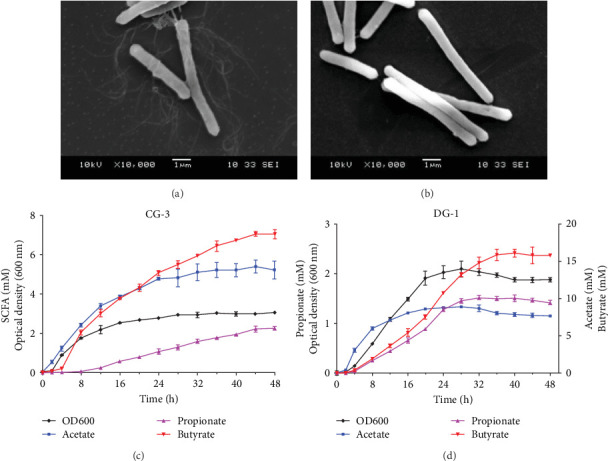
Morphology, growth curve, and SCFAs productions of the two selected bacteria. Electron micrograph of *C. butyricum* CG-3 (A) and *Proteocatella sphenisci* DG-1 (B). Growth curve and SCFAs productions of CG-3 (C) and DG-1 (D) (*n* = 3). Values are presented as mean ± SEM. SCFA, short-chain fatty acid.

Colonies of *C. butyricum* CG-3 (1–4 mm) appeared white, round, or slightly irregular, moist, and shiny on PYAG agar after 24 h at 28°C (Figure S3A). Under the electron microscopic observation, bacterial cells are straight or curved with peritrichous flagella and measured 0.6–0.8 × 3.0–7.0 µm (Figure 2A). Colonies of *P. sphenisci* DG-1 (1–2 mm) appeared milky, with round and smooth edges when grown on PYAG agar (Figure S3B). In scanning electron microscopy, the cells are straight or curved rod-like and measured 0.6–0.8 × 3.0–10.0 µm (Figure 2B).

The growth and capacity of producing SCFAs of *C. butyricum* CG-3 and *P. sphenisci* DG-1 are shown in Figure 2C,D. The growth curves showed that both *C. butyricum* CG-3 and *P. sphenisci* DG-1 reached a stationary phase at 24 h postculture. However, the growth curves of *P. sphenisci* DG-1 appeared to decrease after 32 h postculture, implying reduced growth of bacteria. In the culture of *C. butyricum* CG-3, the production of propionate and butyrate accelerated up to 48 h postculture, while the production of acetate remained unchanged at 32 h postculture. *Proteocatella sphenisci* DG-1 showed the stable production of acetate at 20 h postculture, whereas propionate and butyrate were stable at 32 and 36 h postculture.

The antibiotic susceptibility results showed that *P. sphenisci* DG-1 was sensitive to all tested antibiotics, whereas *C. butyricum* CG-3 was resistant to 3/20 kinds of antibiotics (including amikacin, ceftazidime, and tetracycline) (Table S1).

### 3.3. Growth Performance of *N. coibor*

Growth of *N. coibor* fed different diets in control, CB, PS, CBIG, and PSGS was evaluated after feeding for 8 weeks (Table 2). Compared to the control, CB and CBIG exhibited a significantly improved final weight, and PSGS showed significantly boosted WG (*p*  < 0.05); the SGR in treated groups, except PS, displayed a significant increment (*p*  < 0.05). However, the difference in SR and FCR in CB, PS, CBIG, and PSGS was nonsignificant from the control (*p*  > 0.05).

### 3.4. Intestinal Microbiota Analysis

**Figure 3:**
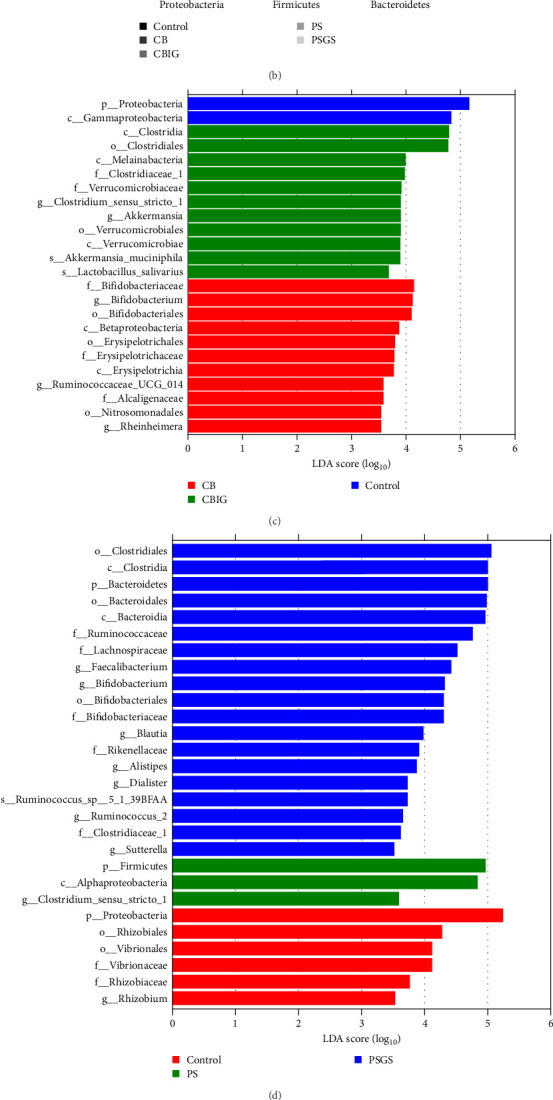
Modulation of gut microbiota by treatment with butyrate-producing bacteria singly or in combination with prebiotics. (A) Alpha diversity indices are shown in the violin plot. (B) Predominant phyla in the intestine. LDA of the intestinal microbiota of *N. coibor* fed control, CB and CBIG diets (C) and control, PS and PSGS diets (D). The alpha value of the Kruskal–Wallis and Wilcoxon test was less than 0.05, and the logarithmic LDA score reached 3.5; the relative abundances of bacterial taxa were considered significant by LEfSe analysis among treatment groups. (E) The discriminatory pathways in the intestine of *N. coibor* fed five different diets. Control (basal diet), CB (basal diet+*C. butyricum* CG3 at 10^7^ CFU/g), CBIG (CB + 0.5% inulin + 0.5% GOS), PS (basal diet+ *P. sphenisci* DG1 at 10^7^ CFU/g), and PSGS (PS + 0.5% GOS + 0.5% D-sorbitol). Values are presented as mean ± SEM. Different superscripts suggest statistically significant differences between diets (*p* < 0.05). GOS, galactooligosaccharides; LDA, linear discriminant analysis.

Totally, sequencing results obtained 1,533,083 reads from 20 samples, with the number of OTUs ranging from 762 to 1698. The rarefaction curves reached a plateau (Figure S4A), suggesting sufficient sequencing depth of bacterial diversity in all samples. Chao1, PD whole tree, and Shannon indices were highest in CB and higher than in the control (*p*  < 0.05) (Figure 3A). Simpson index showed higher values in CB and PSGS than in the control (*p*  < 0.05) (Figures 3A and). PCoA analysis revealed separated and overlapped samples in CB, CBIG, and PS, distinctly clustered with samples in control and PSGS (Figure S4B).

The bacterial phyla Firmicutes, Proteobacteria, and Bacteroidetes were found to predominate in all experimental groups (Figure 3B). The reduced abundance of Proteobacteria in treated groups, except CB and PS, compared to control (*p*  < 0.05). The abundance of Bacteroidetes in PSGS was considerably higher than in the control (*p*  < 0.05). At the genus level, the predominant taxonomic profiles in groups were shown as *Lawaonia* (Control: 7.51%; CB: 0.14%; CBIG: 0.03%; PS: 0.01%; PSGS: 0.02%), unidentified *Chloroplas* (Control: 6.37%; CB: 4.80%; CBIG: 8.90%; PS: 3.07%; PSGS: 1.78%), *Staphylococcus* (Control: 5.73%; CB: 1.90%; CBIG: 1.62%; PS: 8.36%; PSGS: 1.85%), *Bacteroides* (Control: 4.27%; CB: 7.29%; CBIG: 4.65%; PS: 6.36%; PSGS: 15.98%), and *Helicobacter* (Control: 0.03%; CB: 0.22%; CBIG: 4.04%; PS: 0.04%; PSGS: 0.07%) (Figure S4C).

LEfSe analysis revealed that the taxa Proteobacteria and Gammaproteobacteria were abundant in control, whereas Betaproteobacteria, Nitrosomonadales, Erysipelotrichaceae, Alcaligenaceae, Ruminococcaceae UCG_014, *Bifidobacterium*, and *Rheinheimera* were in CB; Melainabacteria, Verrucomicrobiaceae, *Clostridium sensu strict* 1, *Akkermansia muciniphila*, and *Lactobacillus salivarius* were in CBIG (Figure 3C). Moreover, among the control, PS, and PSGS groups, the taxa Proteobacteria, Vibrionales, and *Rhizobium* were enriched in control, while Firmicutes, Alphaproteobacteria, and *Clostridium sensu stricto* 1 were in PS; Bacteroida, Lachnospiraceae, Rikenellaceae, *Faecalibacterium*, *Bifidobacterium*, *Blautia*, *Alistipes*, *Dialister*, *Sutterella*, and *Ruminococcus* sp 5_1_39BFAA were in PSGS (Figure 3D).

The function of microbial communities colonizing the intestines of *N. coibor* in different groups was predicted using the Tax4Fun assignment (Figure 3E). Compared to the control, the two pathways “*Vibrio cholerae* infection” (CBIG, PS, and PSGS) and “Bacterial invasion of epithelial cells” (in CBIG and PSGS) were significantly decreased (*p*  < 0.05) (Figure 3E).

### 3.5. SCFA Formation and Its Association With Abundance of Intestinal Microbes

**Figure 4:**
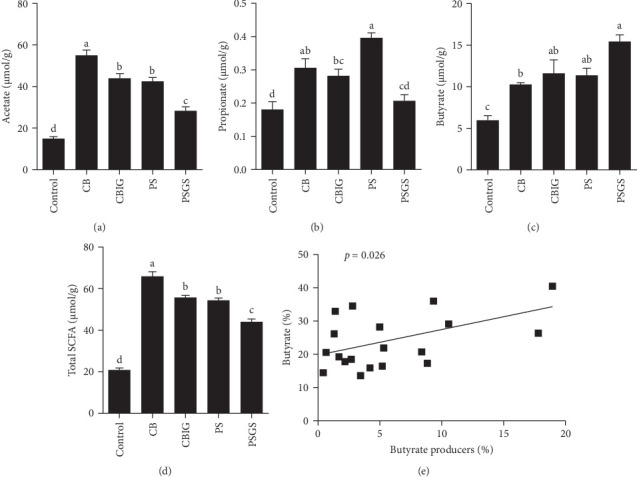
Intestinal SCFAs contents of *N. coibor* fed five different diets and the relationship between the sum of all butyrate-producers and butyrate production. (A) acetate production, (B) propionate production, (C) butyrate production, (D) total SCFA production, (E) relative butyrate production (percentage of total SCFA production) was regressed on the percentage of known BPB (at the genus level, expressed as the percentage of total sequences). Control (basal diet), CB (basal diet+ *C. butyricum* CG3 at 10^7^ CFU/g), CBIG (CB + 0.5% inulin + 0.5% GOS), PS (basal diet+ *P. sphenisci* DG1 at 10^7^ CFU/g), and PSGS (PS + 0.5% GOS + 0.5% D-sorbitol). Values are presented as mean ± SEM. Different superscripts suggest statistically significant differences between diets (*p* < 0.05). BPB, butyrate-producing bacteria; GOS, galactooligosaccharides; SCFAs, short-chain fatty acids.

The content of SCFAs produced in the intestines of fish in experimental groups is presented in Figure 4. Compared to the control, the levels of acetate (in treated groups), propionate (CB, CBIG, and PS), butyrate (treated groups), and total SCFAs (treated groups) were significantly enhanced (Figure 4A–D). Our results proved that the BPB was correlated with butyrate production (*p*=0.026) (Figure 4E).

Fermentation of carbohydrates by gut microbes to produce SCFAs (comprising acetic, propionic, and butyric acids) altered the gut microbial community (Figure S5A). The relative abundance of 14 BPB genera within each experimental group was plotted into a heatmap (Figure S5B). Eight out of these genera, including *Subdoligranulum*, *Anaerostipes*, *Coprococcus*, *Flavonifractor*, *Butyricicoccus*, *Odoribacter*, *Eubacterium*, and *Faecalibacterium*, were more abundant in PSGS than in other groups. The two genera *Acidaminococcus* and *Megasphaera* were predominant in control, while *Roseburia* and *Butyrivibrio* were in PS, and *Oscillibacter* and *Clostridium* were in CBIG. However, the results found that all genera in CB had a lower abundance than other groups (Figure S5B). This finding suggests that different bacteria respond to different carbohydrates with corresponding pathways to produce butyrate.

### 3.6. Physiological and Immunological Responses to Different Diets

**Figure 5:**
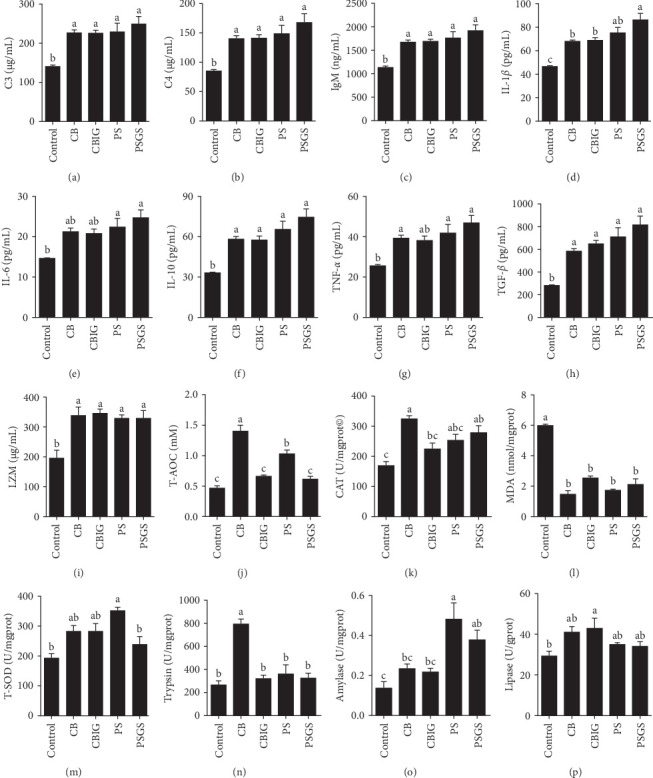
Digestive enzymes, immunity, and antioxidant parameters in the intestine of *N. coibor* fed five different diets. (A) C3, (B) C4, (C) IgM, (D) IL-1β, (E) IL-6, (F) IL-10, (G) TNF-α, (H) TGF-β, (I) LZM, (J) T-AOC, (K) CAT, (L) MDA, (M) T-SOD, (N) Trypsin, (O) Amylase, and (P) Lipase. Control (basal diet), CB (basal diet+ *C. butyricum* CG3 at 10^7^ CFU/g), CBIG (CB + 0.5% inulin + 0.5% GOS), PS (basal diet+ *P. sphenisci* DG1 at 10^7^ CFU/g), and PSGS (PS + 0.5% GOS + 0.5% D-sorbitol). Values are presented as mean ± SEM. Different superscripts suggest statistically significant differences between diets (*p* < 0.05). CAT, catalase; GOS, galactooligosaccharides; IgM, immunoglobulin M; IL, interleukin; LZM, lysozyme; MDA, malondialdehyde; T-AOC, total antioxidant capacity; TGF-β, transforming growth factor-β; TNF-α, tumor necrosis factor alpha; T-SOD, total superoxide dismutase.

After an 8-week feeding period, compared with the control, our results showed an increase in the content of IgM, C3, C4, TGF-*β*, IL-1*β*, IL-10, and LZM, and a decrease of MDA in treated groups (*p*  < 0.05) (Figure 5). Increased values of T-AOC (in CB and PS), T-SOD (PS), CAT (CB and PSGS), TNF-*α* (CB, PS, and PSGS), IL-6 (PS and PSGS), amylase (in PS and PSGS), lipase (CBIG), and trypsin (CB) were obtained by comparison to the control (*p*  < 0.05) (Figure 5).

## 4. Discussion

Our study revealed the high butyrate concentration in the fermentation of *N. coibor* intestinal microbes with inulin or GOS, suggesting that these carbohydrates are suitable substrates to promote butyrate production in the culture medium. Similarly, our investigation demonstrated that a mixture of prebiotics modulates the structure of gut microbiota and their metabolites (SCFAs) to improve growth, activity of antioxidants, and immunity of Chu's croaker [34]. The alternation of gut microbiome and SCFA production, particularly butyrate formation, after supplementation with prebiotics was assessed during in vitro fermentation [12]. Sato et al. [3] mentioned that, in humans, consumption of xylitol or L-sorbose possibly promotes the growth and activity of BPB. Similar to the findings of this study, where the butyrate production was measured to be increased during the in vitro fermentation of gut contents of *N. coibor* supplemented with carbohydrates (inulin or GOS).

Nine isolates identified as *C. butyricum* (six isolates), *P. sphenisci* (two isolates), and *F. varium* (one isolate) were isolated from the fermented cultures of gut contents of *N. coibor* supplemented with either inulin, D-sorbitol, or GOS. Also, the bacteria grew and produced a high concentration of butyrate in both PYL and PYAG media, indicating that these bacterial strains can consume acetate to generate large amounts of butyrate. This finding aligns with the case of *Anaerostipes hadrus* originating from human intestines [3]. Our present study recorded the presence of genes encoding butyryl-CoA:acetate CoA transferase in *C. butyricum* CG-3 and *P. sphenisci* DG-1 genome (unpublished data), indicating that the bacteria can use acetate to boost butyrate production [31, 43]. Furthermore, the findings revealed that *C. butyricum* CG-3 and *P. sphenisci* DG-1 were highly sensitive to antibiotics, indicating that the antimicrobial compounds are rarely used in the culture of *N. coibor*. However, the resistance of *C. butyricum* CG-3 to the antibiotics may suggest that the bacteria carried the natural resistance genes [44]. This was also reported in *Lactobacillus* strains isolated from human saliva, which contains tetracycline resistance (Tet-R) genes in the genome, but these bacteria do not have Tet-R reservoirs [45]. Moreover, Gharbi et al. [46] discussed that ideal probiotic strains should not have risks of transmission of antibiotic-resistance genes and pathogenic properties. This is in agreement with the hemolysis test showing that both *C. butyricum* CG-3 and *P. sphenisci* DG-1 are *γ*-hemolytic bacteria (unpublished data). For these reasons, two bacteria, *C. butyricum* CG-3 and *P. sphenisci* DG-1, may be used as potential probiotics for feed supplementation.

Beyond assessing the butyrate production capability and safety of the candidate strains in vitro, it is even more important to determine their efficacy in fish, such as their ability to improve gut microbiota composition, stimulate the growth of beneficial bacteria, enhance growth performance, hematological parameters, digestive enzyme activity, oxidative status, and immunological parameters [47, 48]. Additionally, a key focus of this study is to compare the effects of BPB singly or in combination with prebiotics.

It has been shown that BPB (i.e., *C. butyricum*) can improve growth, feed utilization efficiency, antioxidant capability, and immunity in fish and shrimps [36]. Our study herein found the increment of WG (PSGS) and SGR (CB, CBIG, and PSGS). However, the growth performance, FCR, and SR of *N. coibor* were positively affected by the single form of BPB or its combination with prebiotics. This is in accordance with cases of fish-fed *C. butyricum* [18, 21, 49, 50]. Feeding fishes with a combination of prebiotics and probiotics improved growth parameters and provided protection against fish pathogens [32, 33, 51]. For instance, *C. butyricum* improved the growth and reduced the FCR of *Miichthys miiuy* [18]. Different results in hybrid grouper were observed with similarity in growth status and digestive enzyme activities of fish fed *C. butyricum* and controls [52]. The observed differences in this study may be attributed to variations in the sources and dosages of probiotics, as well as the species of fish used. Moreover, the combination of BPB with prebiotics (two groups: CBIG and PSGS) revealed a predominant effect on the growth of fish. This agrees with previous findings that prebiotic mixtures of either inulin and GOS or D-sorbitol and GOS increased growth performance in *N. coibor* [34]. Applications of probiotics have been shown to improve growth rates, feed efficiency, and SRs in aquatic animals by influencing digestive enzyme activity [47]. In this study, trypsin activity increased in CB, while lipase increased in CBIG, and amylase increased in PS and PSGS. This indicated that different probiotic bacteria and their combination with prebiotics affect digestive enzyme activity in different ways in *N. coibor*. Similar results were found in *Oreochromis niloticus* and *Oncorhynchus mykiss*-fed diets supplemented with different probiotics [53, 54]. Similarly, *Channa punctatus* fed with combined prebiotic and probiotic supplementation diets showed improved digestibility [51]. An increase in digestive enzyme activity contributes to the improvement in feed efficiency and growth [53]. Collectively, the findings herein proved the contribution of the single use of *C. butyricum* CG-3 and the combination of either *C. butyricum* CG-3 or *P. sphenisci* DG-1 with prebiotics provided better growth of *N. coibor*.

Gut microbiota plays important functions in the host through enhancing nutrient supply, preventing pathogen invasion, maintaining energy homeostasis, and preserving normal mucosal immunity [55]. In fishes, diets, seasonal changes, stress, individual/species variations, gut regions, developmental stages/life cycles, starvation, migration, and water quality are the main factors affecting the structure of the gut microbiome [56]. Previously, Tran et al. [36] pointed out that the dietary addition of *C. butyricum* alters the gut microorganisms of several fish species. Herein, the higher microbial *α*-diversity in CB (i.e., Chao1, PD whole tree, Shannon, and Simpson indexes) and PSGS (Simpson index) was found compared to control. Similarly, the administration of *C. butyricum* or prebiotics promotes the increase of bacterial diversity in the intestines of *O. niloticus* [21] or *S. aurata* [57] and *N. coibor* [34], respectively. The present study found that dietary BPB singly or in combination with prebiotics reduced the growth of Proteobacteria and promoted Firmicutes and Bacteroidetes. This is in accordance with the findings in *O. niloticus* fed with dietary *C. butyricum* [21]. Members of the Bacteroidetes and Firmicutes phyla are known to digest dietary fibers and polyphenols [58], which aligns with this study's results. The decreased abundance of Proteobacteria might contribute to maintaining microbial homeostasis and inhibit pathogen growth. The previous observation revealed that the increase of Proteobacteria is related to dysbiosis during metabolic disorders in the intestines [59]. Herein, functions of microbiota were observed in fish fed BPB singly or in combination with prebiotics. The pathways related to infections, “Bacterial invasion of epithelial cells” (in CBIG and PSGS) and “*V. cholerae* infection” (CBIG, PS, and PSGS), were significantly decreased, indicating an inhibition in the growth of pathogens and, therefore, a reduction of disease outbreaks. Interestingly, more bacterial genera (such as *Clostridium*, *Faecalibacterium*, *Ruminococcus*, *Bacteroides*, *Helicobacter*, and *Bifidobacterium*) were identified in fish administered with BPB singly or in combination with prebiotics than the control group, suggesting the suitable ecosystem for the proliferation of these bacteria. It has been shown that the growth of individual bacterial species was based on the kinds of carbohydrate supplemented [43]. Additionally, concentrations of SCFAs significantly increased in CB, CBIG, PS, and PSGS, indicating the selective growth of SCFA-producing bacteria in these treated groups. This is also confirmed by strong associations of several bacteria with concentrations of SCFAs. These results indicated the functions of BPB singly or in combination with prebiotics in maintaining gut microbial homeostasis and promoting SCFA production.

Supplementation of probiotics in the diet is considered a crucial approach to enhance the immunity of fishes [60]. LZM activity and levels of IgM, proinflammatory cytokines, and complement components have been widely used for evaluating the responses of fish to the supplementation of probiotics or prebiotics [34, 60, 61]. We found that C3, C4, IgM, IL-1*β*, IL-10, TGF-*β*, and LZM contents were boosted in treated groups. Similar situations were recorded in *Epinephelus coioides* fed diets implemented with lactic acid bacteria [62], *O. niloticus* fed with a commercial product comprising *Bacillus subtilis* and *Bacillus licheniformis* [63], *Cyprinus carpio* fed with *Enterococcus casseliflavus* (EC-001) [60], and *N. coibor* fed with prebiotic mixtures [34]. The increased levels of C3 and C4, and LZM indicated that the BPB singly or in combination with prebiotics may stimulate the activation of the complement cascade and the activity of antimicrobial components [60]. T-AOC, CAT, and SOD are antioxidative biomarkers, as well as MDA is a prooxidant biomarker [64]. Herein, the increase of T-AOC (in the CB and PS), CAT (in the CB and PSGS), and T-SOD (in the PS) and the decrease of MDA were noted in treated groups. Similar observations were made in *O. niloticus* fed *C. butyricum* [21]. Some previous studies have confirmed that supplementation with a combination of prebiotics and probiotics increased antioxidant levels (SOD, CAT, MDA, glutathione peroxidase [GPx], myeloperoxidase [MPO]), specific and nonspecific immune parameters such as respiratory burst (RB), reactive oxygen species (ROS), IgM, acid phosphatase (ACP), phagocytic activity (PA), alternative complement pathway (ACP), pro- and anti-inflammatory markers (C3, C4, IL-1*β*, IL-8, IL-10, TGF-*β*, TNF-*α*, NF-*κ*B, iNOS) in *H. molitrix*, *Labeo rohita*, and *Channa punctatus* [32, 33, 51]. Thus, *C. butyricum* CG-3 or *P. sphenisci* DG-1 alone and the combination of BPB with prebiotics could affect the immune response of *N. coibor* through the activation of antioxidant activities.

Probiotics and synbiotics have been recognized for their immunostimulatory properties associated with regulating the synthesis of inflammatory cytokines [65]. Cytokine expressions exhibit the regulation of immune activity and, therefore, protect the host against infections [54]. Expressions of TNF-*α*, TGF-*β*, IL-6, and IL-1*β* increased in fish administered by probiotics or synbiotics [54, 66]. These cytokines are involved in promoting inflammatory responses in the intestine during bacterial colonization or invasion [67]. The cytokines IL-1*β* and TNF-*α* expressed during the early stages of immunity activation, while TGF-*β* acts as a dual-function molecule with inhibitory and stimulatory effects [67]. IL-6 is involved in inflammation in response to infection and the regulation of regenerative, metabolic, and neural processes [68]. Herein, the increased expression of TNF-*α* (in the CB, PS, and PSGS) and IL-6 (PS and PSGS) was found when compared to the control, suggesting that the BPB singly or in combination with prebiotics can regulate the activation of inflammatory cytokines, subsequently protecting the host from excessive inflammation. Similarly, previous studies have shown an increment in the level of TGF-*β*, TNF-*α*, IL-6, and IL-1*β* [54] and TLR2, MyD88, IRAK-4, TNF-*α*, and IL-8 genes [21] in *O. niloticus* fed with *Rummeliibacillus stabekisii* and *C. butyricum*, respectively. Dietary prebiotics can stimulate the expressions of TGF-*β*, TNF-*α*, IL-6, IL-10, and IL-1*β* in *N. coibor* [34]. Collectively, *C. butyricum* CG-3 or *P. sphenisci* DG-1 alone and in combination with prebiotics could improve the immune response of *N. coibor* by stimulating antioxidant enzyme activation and pro-inflammatory cytokine expression. However, the obtained results need to be supported by more evidence, such as the simultaneous application of BPB singly or in combination with prebiotics and a challenge test with pathogen-caused diseases in *N. coibor*.

## 5. Conclusion

In conclusion, BPB (with the presentative isolates *C. butyricum* CG-3 and *P. sphenisci* DG-1) were isolated from the in vitro fermentation using gut contents of *N. coibor*, indicating that this approach can be used as a strategy for the isolation of BPB. The feeding experiments proved that BPB singly or in combination with GOS and inulin or D-sorbitol can boost the growth and immunity of *N. coibor*. Furthermore, dietary administration of BPB singly or in combination with GOS and inulin or D-sorbitol can select the intestinal microbiota (especially butyrate producers), maintain gut homeostasis, and enhance the concentration of SCFAs in *N. coibor*. Our findings suggest that BPB can be used in *N. coibor* farming.

## Figures and Tables

**Table 1:** The identification and butyrate production of nine isolates.

Strain	LDCs	Butyrate in PYL broth (mM)*⁣*^*∗*^	Butyrate in PYAG broth (mM)*⁣*^*∗*^	Similar species (% identity)
CI-1	Inulin	4.75 ± 0.48	22.88 ± 0.62	*C. butyricum* JCM 1391 (99.93%)
CI-2	Inulin	5.27 ± 0.33	26.05 ± 0.51	*C. butyricum* JCM 1391 (99.30%)
CG-1	GOS	17.12 ± 0.66	21.41 ± 2.10	*C. butyricum* JCM 1391 (99.93%)
CG-2	GOS	12.13 ± 1.85	24.90 ± 0.67	*C. butyricum* JCM 1391 (99.79%)
CG-3	GOS	18.90 ± 2.56	26.50 ± 0.85	*C. butyricum* JCM 1391 (100%)
CG-4	GOS	12.98 ± 0.13	24.89 ± 1.10	*C. butyricum* JCM 1391 (99.93%)
CG-5	GOS	9.84 ± 0.61	21.38 ± 0.50	*F. varium* JCM 6320 (99.72%)
DG-1	GOS	4.35 ± 0.22	27.76 ± 0.28	*Proteocatella sphenisci* PPP2 (97.87%)
CS-1	D-sorbitol	4.03 ± 0.32	13.03 ± 0.41	*Proteocatella sphenisci* PPP2 (98.17%)

Abbreviation: GOS, galactooligosaccharides.

*⁣* ^*∗*^Values are presented as mean ± SEM.

**Table 2:** Growth parameters of *N. coibor* fed different diets after 8 weeks of feeding.

Parameter	Control	CB	CBIG	PS	PSGS
Initial weight (g)	13.58 ± 0.07	13.77 ± 0.12	13.77 ± 0.31	13.60 ± 0.28	13.2 ± 0.06
Final weight (g)	36.61 ± 0.43^b^	41.18 ± 0.98^a^	41.15 ± 0.40^a^	39.77 ± 1.40^b^	39.90 ± 0.79^b^
Weight gain (WG) (%)	169.6 ± 4.4^b^	199.3 ± 9.67^b^	199.1 ± 4.21^b^	192.3 ± 6.17^b^	202.4 ± 7.17^a^
Specific growth rate (SGR)	1.77 ± 0.03^b^	1.96 ± 0.06^a^	1.96 ± 0.03^a^	1.92 ± 0.04^b^	1.975 ± 0.04^a^
Feed conversion ratio (FCR)	1.34 ± 0.08	1.25 ± 0.08	1.29 ± 0.01	1.25 ± 0.09	1.23 ± 0.03
Survival rate (SR)	88.33 ± 1.67	96.67 ± 1.67	95 ± 2.89	88.33 ± 3.33	93.33 ± 1.67

*Note:* Total fish biomass data from each treatment (three replicate cages per treatment, 20 fish per cage) were used to calculate the above growth parameters. Control (basal diet), CB (basal diet+ *C. butyricum* CG3 at 10^7^ CFU/g), CBIG (CB + 0.5% inulin + 0.5% GOS), PS (basal diet+ *P. sphenisci* DG1 at 10^7^ CFU/g), and PSGS (PS + 0.5% GOS + 0.5% D-sorbitol). Values are presented as mean ± SEM. Different superscripts suggest statistically significant differences between diets (*p* < 0.05).

Abbreviation: GOS, galactooligosaccharides.

## Data Availability

The full-length 16S rRNA gene sequences of the two strains obtained in this study were submitted to GenBank (accession numbers: PQ614736–PQ614737). The 16S rRNA gene V4 region sequences of the gut microbiome were submitted to the NCBI Sequence Read Archive (SRA) under accession number SRP379037.

## Supporting Information

Supporting Information Table S1: antibiotic susceptibilities of two representative bacterial isolates, *Clostridium butyricum* CG-3 and *Proteocatella sphenisci* DG-1. Figure S1: pre-screening of eight kinds of carbohydrates that promoted butyrate formation in the in vitro cultures. Figure S2: phylogenetic tree of nine butyrate-producing bacteria. Figure S3: colonial morphology of strain *C. butyricum* CG-3 and *P. sphenisci* DG-1. Figure S4: the composition of gut microbiota is changed by treatment with butyrate-producing bacteria and the combination of which with prebiotics. Figure S5: overview of known SCFA-producing bacteria in major SCFA formation pathways, and the distribution of butyrate-producing bacteria in the intestine of *N. coibor*.
