# Supplementary material for: Isolation and Evaluation of Potential Use of Prebiotics—Utilizing Butyrate-Producing Bacteria in Nibea coibor
Source: Aquac Nutr. 2025 Jan 11;2025:4679037. doi: 10.1155/anu/4679037 (PMC11742079; doi:10.1155/anu/4679037)
Supplement: Supporting Information — Table S1: antibiotic susceptibilities of two representative bacterial isolates, Clostridium butyricum CG-3 and Proteocatella sphenisci DG-1. Figure S1: pre-screening of eight kinds of carbohydrates that promoted butyrate formation in the in vitro cultures. Figure S2: phylogenetic tree of nine butyrate-producing bacteria. Figure S3: colonial morphology of strain C. butyricum CG-3 and P. sphenisci DG-1. Figure S4: the composition of gut microbiota is changed by treatment with butyrate-producing bacteria and the combination of which with prebiotics. Figure S5: overview of known SCFA-producing bacteria in major SCFA formation pathways, and the distribution of butyrate-producing bacteria in the intestine of N. coibor. [file 4679037.f1.docx]

**Supplemental Materials**

**Table S**

**TABLE S1** Antibiotic susceptibilities of two representative bacterial isolates, *Clostridium butyricum* CG-3 and *Proteocatella sphenisci* DG-1

| Antibiotic | CG-3 | DG-1 | Antibiotic | CG-3 | DG-1 |
| --- | --- | --- | --- | --- | --- |
| Amikacin | R | S | Doxycycline | S | S |
| Ampicillin | S | S | Erythromycin | S | S |
| Carbenicillin | S | S | Gentamicin | S | S |
| Cefalexin | S | S | Kanamycin | S | S |
| Cefazolin | S | S | Minocycline | S | S |
| Cefoperazone | S | S | Neomycin | S | S |
| Cefradine | S | S | Oxacillin | S | S |
| Ceftriaxone | S | S | Penicillin | S | S |
| Ceftazidime | R | S | Piperacillin | S | S |
| Cefuroxime | S | S | Tetracycline | R | S |

R, Resistant; I, Intermediate; S, Susceptible.

**Figure legends**

**FIG S1** Pre-screening of eight kinds of carbohydrates that promoted butyrate formation in the *in vitro* cultures. Cultures were prepared from intestinal contents collected from *N. coibor* (n=3). Values are presented as mean±SEM.

**FIG S2** Phylogenetic tree of nine butyrate-producing bacteria. A Neighbor-Joining tree was constructed, and branch width changed according to bootstrap values (expressed as a percentage of 1000 replications).

**FIG S3** Colonial morphology of strain *Clostridium butyricum* CG-3 (A) and *Proteocatella sphenisci* DG-1 (B)

**FIG S4** The composition of gut microbiota is changed by treatment with butyrate-producing bacteria and the combination of which with prebiotics. (A) The rarefaction curves present the relationship between sequence number and observed species number. (B) Binary-Jaccard PCoA plot based on OTU abundance. Each point represents a sample and the colors represent the different treatments. (C) Predominant genera in the intestine of *Nibea coibor* fed five different diets. Control (basal diet), CB (basal diet+*C. butyricum* CG3 at 10^7^ CFU/g), CBIG (CB+0.5% inulin+0.5% GOS), PS (basal diet+ *P. sphenisci* DG1 at 10^7^ CFU/g), and PSGS (PS+0.5% GOS+0.5% D-sorbitol).

**FIG S5** Overview of known SCFA-producing bacteria in major SCFA formation pathways (A), and the distribution of butyrate-producing bacteria in the intestine of *N. coibor* (B). Control (basal diet), CB (basal diet+*C. butyricum* CG3 at 10^7^ CFU/g), CBIG (CB+0.5% inulin+0.5% GOS), PS (basal diet+ *P. sphenisci* DG1 at 10^7^ CFU/g), and PSGS (PS+0.5% GOS+0.5% D-sorbitol).

**Figures**

**FIG S1**


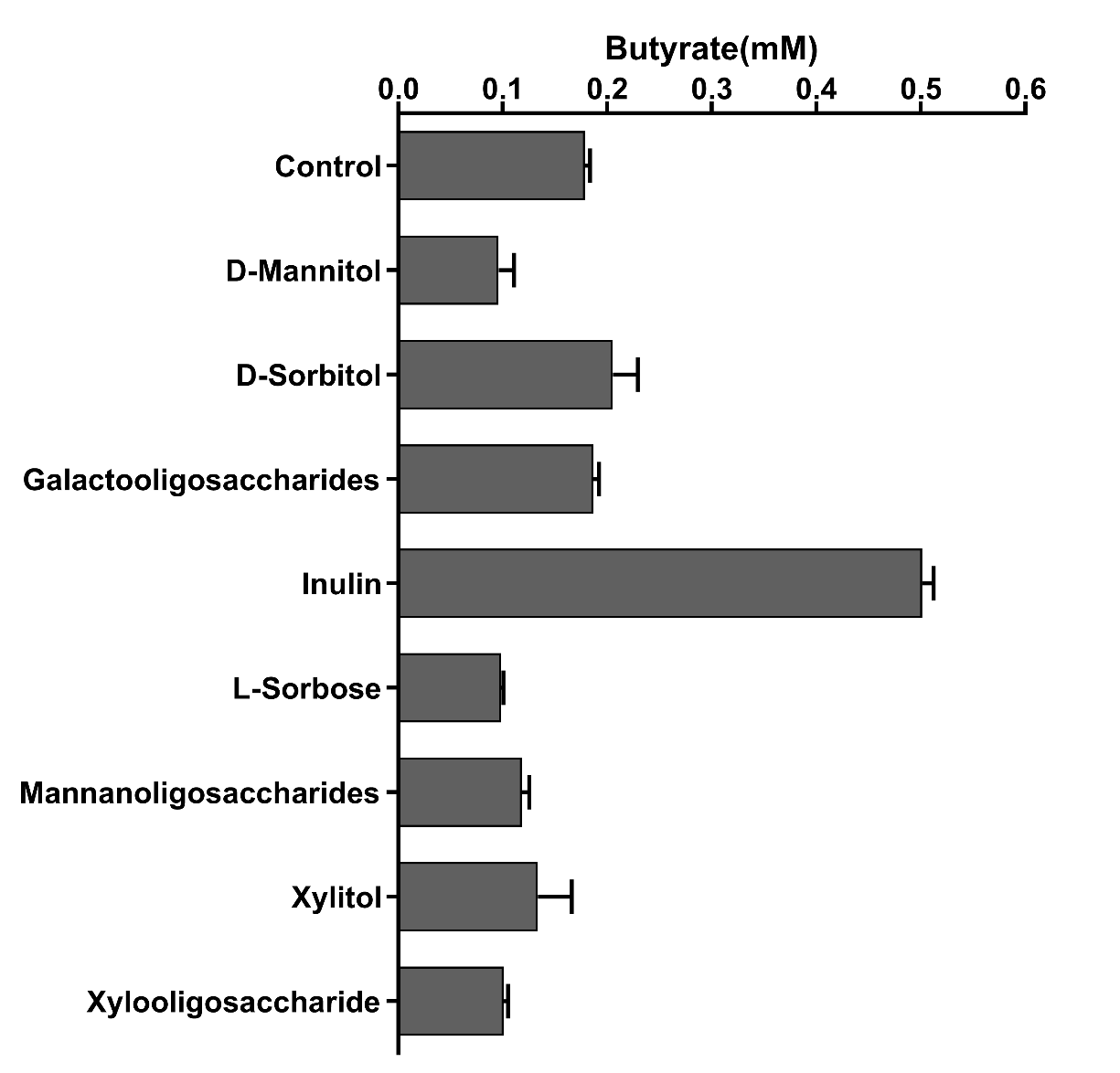


**FIG S2**

**FIG S3**

**FIG S4**

**FIG S5**
